# Potential of functional flavonoids in targeting vasospasm through modulation of oxidative stress and SPC-induced signaling pathways

**DOI:** 10.3389/fphar.2025.1594060

**Published:** 2025-06-11

**Authors:** Ying Zhang, Sho Maejima, Kentaro Matsuzaki, Hiroko Kishi

**Affiliations:** Department of Environmental Physiology, Faculty of Medicine, Shimane University, Izumo, Shimane, Japan

**Keywords:** flavonoids, vasospasm, oxidative stress, SPC, ROS

## Abstract

Vasospasm is a sustained abnormal contraction of vascular smooth muscle (VSM), which is commonly observed in the coronary and cerebral arteries. This abnormal VSM contraction leads to reduced blood flow to tissues or organs, ultimately causing severe diseases such as myocardial infarction and cerebral infarction. Studies have demonstrated that oxidative stress and sphingosylphosphorylcholine (SPC)-induced Rho-kinase signaling pathways are related to this abnormal contraction. Flavonoids, a class of natural compounds, are found in various plants, fruits, vegetables, and traditional Chinese medicines. They have anti-inflammatory, antioxidative, and anticarcinogenic properties. Recent studies have shown that some flavonoids strongly inhibit the abnormal contraction of VSM. This review explores the potential of flavonoids as candidate drugs for the treatment and prevention of vasospasm through oxidative stress and the SPC-induced Rho-kinase signaling pathway. Nevertheless, more extensive studies are required to fully elucidate the mechanism by which flavonoids exert their anti-vasospastic effects and explore their potential benefits as adjunctive therapy for critical cardiovascular and cerebrovascular diseases.

## 1 Introduction

Vasospasm, such as coronary vasospasm and cerebral vasospasm, is the abnormal contraction of vascular smooth muscle (VSM) leading to blood vessel narrowing. This narrowing reduces blood flow, resulting in serious cardiovascular and cerebrovascular diseases, such as myocardial infarction, angina, and cerebral infarction (Hung et al., 2014; Slavich and Patel, 2016; Maruhashi and Higashi, 2021; Sinha et al., 2022). The underlying pathophysiology of vasospasm involves multiple factors, including endothelial dysfunction, autonomic nervous system imbalances, vascular smooth muscle hypercontractility, and activated signaling pathway of vasoactive mediators (Kolias et al., 2009; Matta et al., 2020). Although the pathogenesis of vasospasm has not been fully elucidated, endothelial dysfunction and increased VSM contractility are considered to be the main underlying mechanisms (Kusama et al., 2011; Franczyk et al., 2022). Oxidative stress plays an important role in vasospasm by promoting endothelial dysfunction, activating vasoconstrictors such as endothelin-1 (ET-1), and impairing the nitric oxide (NO) vasodilator system (Ruef et al., 2001; Higashi et al., 2009; Higashi, 2022). Sphingosylphosphorylcholine (SPC) is an active sphingolipid that induces the Rho-kinase signaling pathway involved in abnormal contraction of VSM (Todoroki-Ikeda et al., 2000; Shirao et al., 2002). So far, there is no effective treatment for vasospasm. Therefore, understanding the underlying mechanism of vasospasm and finding new therapeutic strategies are crucial for treating vasospasm-associated diseases.

Recent studies have found that natural compounds with antioxidant properties can reduce oxidative stress and improve vascular function (Mukherjee et al., 2024). Among them, flavonoids, widely distributed in plants and traditional Chinese medicine herbs, have shown promise in protecting against oxidative damage, restoring vascular homeostasis, and preventing vasospasm (Xu et al., 2022; Li R. L. et al., 2023). Some evidence suggests that flavonoids reduce the risk of cardiovascular diseases and have cardiovascular protective effects (Erdman et al., 2007; Horakova, 2011). Quercetin, kaempferol, and catechins can relax the smooth muscle of the coronary artery, thereby reducing the incidence of coronary vasospasm (Xu et al., 2015; Mangels and Mohler, 2017; Dagher et al., 2021). Flavonoids have also been found to improve endothelial function and reduce mortality of cardiovascular diseases (Perez-Vizcaino et al., 2006; Yamagata and Yamori, 2020). In addition, they contribute to regulate cholesterol levels, lower blood pressure, and reduce the risk of thrombosis (Ciumarnean et al., 2020; Kozlowska and Szostak-Wegierek, 2022). A recent study showed that flavonoids effectively prevent brain damage caused by intracerebral hemorrhage and subarachnoid hemorrhage (SAH) by inhibiting inflammation and oxidative stress (Dong et al., 2024). Furthermore, hesperetin and tangeretin significantly inhibit SPC-induced abnormal contraction (Li et al., 2022; Lu et al., 2022).

In recent years, there has been an increasing interest in how oxidative stress affects vascular function. At the same time, antioxidants have been widely used as adjuvant therapy for a variety of clinical diseases. A deeper understanding of the mechanisms regulating abnormal vascular contraction is essential for further revealing the pathophysiology of cardiovascular and cerebrovascular diseases. This review comprehensively explores the mechanism of action of oxidative stress and SPC in vasospasm and summarizes the research progress on the regulation of vascular tension by natural flavonoids. This article aims to promote in-depth research in this field and provide effective intervention strategies for improving and preventing coronary artery spasm and cerebral vasospasm.

## 2 Roles of oxidative stress and SPC in vasospasm

### 2.1 Role of oxidative stress in vasospasm

Oxidative stress is a state of imbalance between the generation and accumulation of reactive oxygen species (ROS) and the clearance of these ROS in cells and tissues (Vona et al., 2021). Under pathological conditions, such as brain injury and SAH, oxidative stress impairs endothelial function and increases the production of ROS during hemoglobin degradation, mitochondrial dysfunction, and disrupted antioxidant systems (Ayer and Zhang, 2008; Hao et al., 2022). These processes result in vasoconstriction, increased vascular resistance, and a propensity for vasospasm. Studies have demonstrated that oxidative stress contributes to cerebral vasospasm after SAH (Macdonald and Weir, 1994; Kim et al., 2002; Wu F. et al., 2021; Hao et al., 2022). Increased levels of superoxide anions in the cerebrospinal fluid following SAH have been reported to be associated with cerebral vasospasm (Fumoto et al., 2019). In animal models of SAH, accumulating evidence has shown that free radical scavengers, such as iron chelators, ebselen, U74006F, and inhibitors of free radical-generating enzymes attenuate cerebral vasospasm (Matsui and Asano, 1994; Watanabe et al., 1997; Horky et al., 1998; Handa et al., 2000; Zheng et al., 2005). Maeda Y et al. demonstrated that oxidative stress significantly impairs bradykinin-induced, endothelium-dependent relaxation in bovine middle cerebral arteries. (Maeda et al., 2004). In addition, oxidative stress contributes to smooth muscle cell proliferation and hypertrophy, as well as endothelial cell apoptosis (Satoh et al., 2010). Research is ongoing to further elucidate how oxidative stress affects cerebral vasoconstrictor responses and contributes to the development of vasospasm.

### 2.2 Oxidative stress-related signaling pathways in vasospasm

**FIGURE 1:**
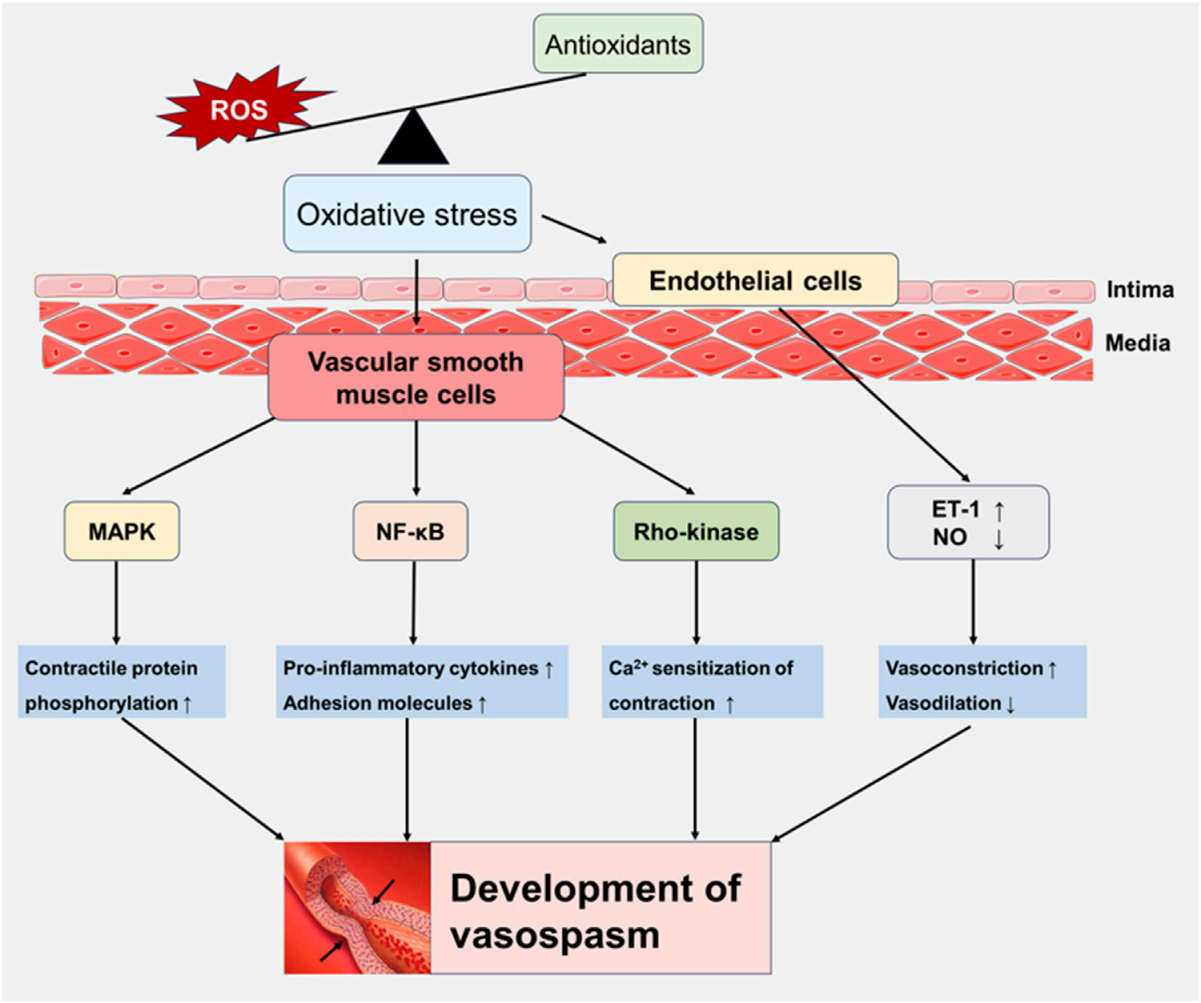
Oxidative stress-induced signaling pathways involved in vasospasm. Oxidative stress damages endothelial cell function and increases the expression of endothelin-1 (ET-1) by increasing the generation of reactive oxygen species (ROS), while inhibiting the vasodilator factor NO, increasing vasoconstriction and weakening vasodilation. In addition, ROS activate key signaling pathways of vascular smooth muscle cells, including NF-κB, Rho-kinase, and MAPK, leading to inflammation and vascular dysfunction, playing a key role in development of vasospasm.

Oxidative stress activates multiple molecular pathways that regulate vascular tone, playing a crucial role in the development of vasospasm. The production of ROS during oxidative stress damages endothelial cells, leading to endothelial dysfunction and impairing the endothelium’s ability to produce vasodilators such as NO (Shaito et al., 2022), which contributes to vasoconstriction and the development of vasospasm. Additionally, ROS enhance the expression of ET-1, and elevated ET-1 levels are closely associated with vasospasm (Saitoh et al., 2009). ROS also activate NF-κB, which triggers the expression of pro-inflammatory cytokines and adhesion molecules, further exacerbating endothelial dysfunction and vascular constriction (Scioli et al., 2020). Increased oxidative stress can also activate the RhoA/Rho-kinase signaling pathway (de Souza et al., 2016), a key factor in the pathophysiology of cerebral vasospasm after SAH (Naraoka et al., 2013). This activation, along with mechanical stress and hypotonic conditions, influences paxillin phosphorylation and RhoA translocation (Lopez-Colome et al., 2017). Accumulating studies have emphasized Fyn expression is upregulated and activated by oxidative stress (Gao et al., 2009; Giannoni and Chiarugi, 2014; Matsushima et al., 2016). Additionally, ROS activate the mitogen-activated protein kinase (MAPK) pathway, leading to the phosphorylation of proteins that promote vasoconstriction and inflammation (Higashi, 2022). These findings demonstrate that oxidative stress triggers various molecular pathways and activates vasoconstriction-related proteins, which play an important role in the onset and progression of vasospasm (Figure 1).

Additionally, increasing evidence indicates that mitochondrial dysfunction–induced oxidative stress may play a critical role in the development of vasospasm (Jacobsen et al., 2014; Zhang et al., 2022). Mitochondria are essential for cellular energy metabolism and redox homeostasis, and mitochondrial dysfunction can lead to oxidative stress, impaired bioenergetics, and vascular dysregulation (Zong et al., 2024). Mitochondria are the main source of ROS, mainly produced in complexes I and III of the electron transport chain (Angelova and Abramov, 2018; Zhao R. Z. et al., 2019). Under pathological conditions, impaired mitochondrial dynamics lead to elevated mitochondrial ROS, which in turn reduces the bioavailability of NO by reacting with it to form peroxynitrite (Prajapat et al., 2024), ultimately causing impaired endothelium-dependent vasodilation and contributing to the occurrence of vasospasm. Increased mitochondrial ROS and calcium overload in cerebral arteries of SAH patients suggest that they may aggravate delayed cerebral vasospasm (Zhang et al., 2022). In addition, oxidative stress induced by mitochondrial dysfunction leads to reduced ATP synthesis (Bhatti et al., 2017), which in turn affects the sarcoplasmic or endoplasmic reticulum Ca^2+^-ATPase (SERCA) pump’s ability to actively transport Ca^2+^ from the cytosol into the lumen of the sarcoplasmic reticulum and endoplasmic reticulum (Wu et al., 2001), a process that is critical for maintaining low cytoplasmic calcium levels and promoting muscle relaxation, thereby impairing vascular tone regulation and possibly inducing vasospasm.

### 2.3 The role of SPC in vasospasm

Sphingosylphosphorylcholine (SPC) is a naturally occurring bioactive sphingolipid in blood plasma that has emerged as an important modulator of cardiovascular functions (Ge et al., 2018). Under physical conditions, circulating SPC levels are about 50 nM in plasma and 130 nM in serum (Liliom et al., 2001). SPC acts both as an extracellular first messenger via G protein-coupled receptors (such as S1P1–3 and GPR12) (Meyer zu Heringdorf and Jakobs, 2007; Ge et al., 2018) and as an intracellular second messenger by directly regulating the ryanodine receptor in cardiomyocytes (Uehara et al., 1999; Yasukochi et al., 2003). In the heart, SPC regulates Na^+^ and Ca^2+^ currents (Yasui and Palade, 1996) and protects cardiomyocytes against ischemia/reperfusion-induced apoptosis and inflammation through autophagy mediated by the lipid raft/PTEN/Akt/mTOR pathway (Yue et al., 2015). In vascular endothelial cells, SPC at low concentrations (≤10 μM) exhibits anti-apoptotic and anti-inflammatory effects (Ge et al., 2011), whereas at higher concentrations (≥10 μM), it may induce oxidative stress and inflammation (Jeon et al., 2007). In vascular smooth muscle cells, SPC promotes cell migration (Boguslawski et al., 2002; Zhang et al., 2021) and Ca^2+^ sensitization through the Src/Rho-kinase pathway (Nakao et al., 2002), contributing to vascular remodeling and vasospasm. Furthermore, SPC induces the differentiation of mesenchymal stem cells into smooth muscle-like cells (Jeon et al., 2006), suggesting a potential role in vascular repair. In summary, SPC exerts multifaceted effects on the cardiovascular system. In this review, we focus on the role of SPC in the abnormal contraction of vascular smooth muscle.

Under normal physiological conditions, VSM contraction plays a crucial role in maintaining blood pressure, blood flow, and vascular tone. This contraction is dependent on Ca^2+^ (Somlyo and Somlyo, 1994) and can be triggered by mechanical, electrical, or chemical stimuli through the Ca^2+^/calmodulin (CaM)-myosin light chain kinase (MLCK) signaling pathway (Kamm and Stull, 1985). When vascular smooth muscle cells are stimulated, the intracellular Ca^2+^ concentration increases, binding to CaM to form a Ca^2+^/CaM complex. This complex induces a conformational change in MLCK, thereby activating it (Kemp and Pearson, 1991). Activated MLCK then phosphorylates myosin light chain (MLC), promoting the formation of actin-myosin crossbridges, leading to muscle contraction (Kamm and Stull, 1985). However, the Ca^2+^-independent mechanism has also been reported to contribute to VSM contraction. The pathways involved including RhoA-Rho-kinase (Kureishi et al., 1997; Mizuno et al., 2008), protein kinase C (Walsh et al., 1996; Dimopoulos et al., 2007), MAPK signaling (Cain et al., 2002), and ROS (Jin et al., 2004). These signals ultimately phosphorylate myosin phosphatase targeting subunit 1 (MYPT1), a subunit of myosin light chain phosphatase (MLCP), reducing MLCP activity and preventing the dephosphorylation of MLC (Alvarez-Santos et al., 2020). As a result, MLC remains in a sustaining phosphorylated state, promoting actin-myosin crossbridge formation and muscle contraction, independent of calcium ion (Webb, 2003; Hirano, 2007). Vasospasm is considered a pathological condition characterized by sustained vascular hyperresponsiveness or Ca^2+^-sensitization of VSM contraction (Tanaka et al., 1998; Dimopoulos et al., 2007). SPC has been identified as a key bioactive lipid mediator that activates Rho-kinase and plays a central role in vasospasm. A study by Kurokawa T et al. showed that SPC concentration is significantly elevated in the cerebrospinal fluid of patients with cerebral vasospasm (Kurokawa et al., 2009). Additionally, injecting SPC into the cisterna magna of the cerebellum and medulla oblongata induces significant and prolonged vasospasm in the canine basilar artery (Shirao et al., 2008). Furthermore, SPC has been shown to induce smooth muscle contraction in various vascular tissues, including the cerebral and coronary arteries, as well as human coronary artery smooth muscle cells (Shirao et al., 2002; Zhang et al., 2017; Li et al., 2022; Lu et al., 2022; Zhang et al., 2024).

### 2.4 The SPC-induced signaling pathway involved in vasospasm

Early studies have demonstrated that the Rho-kinase pathway is significantly activated in cerebral arterial smooth muscle during cerebral vasospasm, a severe complication following SAH (Sato M. et al., 2000; Wang et al., 2014; Hu et al., 2023). This finding suggests that Rho-kinase plays a crucial role in the pathogenesis of vasospasm, contributing to sustained smooth muscle contraction. Shirao S et al. reported that SPC activates Rho-kinase, leading to Ca^2+^-independent contraction in bovine cerebral artery VSM strips (Shirao et al., 2002). A specific Rho-kinase inhibitor, Y27632, along with a dominant-negative Rho-kinase construct, effectively abolished the abnormal contraction of VSM strips induced by SPC (Shirao et al., 2002). Furthermore, SPC stimulation also triggers the translocation of Rho-kinase from the cytoplasm to the cell membrane in VSM cells (Zhang et al., 2017; Li et al., 2022; Lu et al., 2022). These results suggest that Rho-kinase is a downstream molecule in the SPC-induced signaling pathway, which is involved in abnormal contraction.

Fyn is a member of the Src family of non-receptor tyrosine kinases, which plays an important role in cellular signaling, including cell growth, differentiation, and motility (Peng and Fu, 2023). Fyn is also involved in several pathways that regulate smooth muscle contraction, and recent studies have highlighted its role in mediating SPC-induced abnormal contraction in vascular smooth muscle cells (VSMCs). Inhibition of Fyn activity using specific Fyn inhibitors decreased the contraction of VSM strips exposed to SPC, highlighting the importance of Fyn in mediating SPC-induced VSM contraction (Nakao et al., 2002; Lu et al., 2022). Further study found that specific Fyn inhibitors such as PP1 and EPA blocked the activation and translocation of Rho-kinase (Nakao et al., 2002). These findings collectively suggest that Fyn and Rho-kinase play a critical role in mediating abnormal contraction of VSM induced by SPC.

Paxillin, a scaffolding protein located at focal adhesion, recruits various signaling molecules including Fyn and FAK, playing an important role in cytoskeletal reorganization (Singh et al., 2019; Zhang et al., 2021; Zhang et al., 2023). Several studies have shown that paxillin regulates the Ca^2+^-dependent contraction in tracheal smooth muscle and cardiac contractility (Hirth et al., 2016; Zhang W. et al., 2016). Recent study reported that paxillin is a binding molecule of the active Fyn (Zhang et al., 2021). Paxillin deletion attenuates the SPC-induced contraction of VSM, suggesting that paxillin is involved in the SPC-induced abnormal contraction of VSM (Zhang et al., 2024). In addition, the activity of Rho-kinase but not Fyn is inhibited in paxillin deleted cells and tissues (Zhang et al., 2024), indicating that paxillin involves in the SPC-induced abnormal contraction as a signaling molecule between Fyn and Rho-kinase.

**FIGURE 2:**
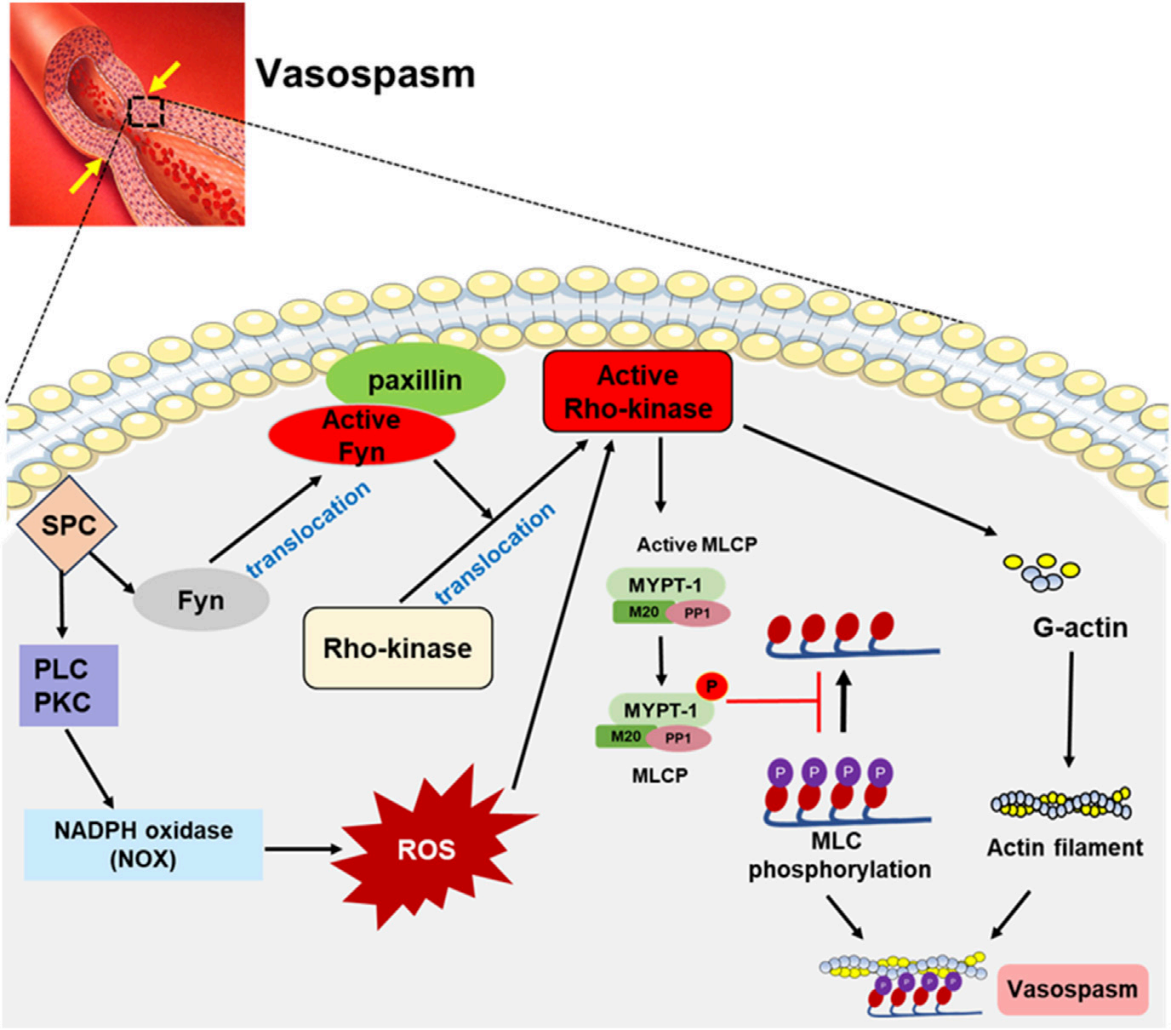
SPC-induced signaling pathways involved in vasospasm. SPC-mediated signaling pathway involves Fyn, a Src family kinase, which promotes Rho-kinase activation by interacting with paxillin. In addition, SPC can activate NADPH oxidase (NOX) through PLC and PKC, increasing ROS generation, which activates the Rho-kinase. The activated Rho-kinase phosphorylates myosin light chain phosphatase (MLCP) to inactivate it, and myosin light chain (MLC) remains in a highly phosphorylated state, leading to vasospasm.

In addition, SPC increases ROS generation by activating PLC, PKC1, and Src-dependent NADPH oxidase 1 (NOX1), thereby enhancing Ca^2+^ entry through L-type channels and strongly enhancing vascular reactivity (Shaifta et al., 2015). Jin L et al. demonstrated that ROS-induced vascular contraction is mediated through the activation of the Rho/Rho-kinase pathway, as evidenced by the increased translocation of Rho to the membrane and phosphorylation of MYPT1, both of which were inhibited by the Rho-kinase inhibitor Y-27632 (Jin et al., 2004). SPC induces apoptosis of endothelial cells through ROS-mediated activation of ERK, causing endothelial dysfunction (Jeon et al., 2007). These studies suggest that SPC could impact vascular contraction activity through ROS-mediated pathways. The signaling pathways induced by SPC are summarized as shown in Figure 2.

## 3 Anti-vasospasm effect of flavonoids

**FIGURE 3:**
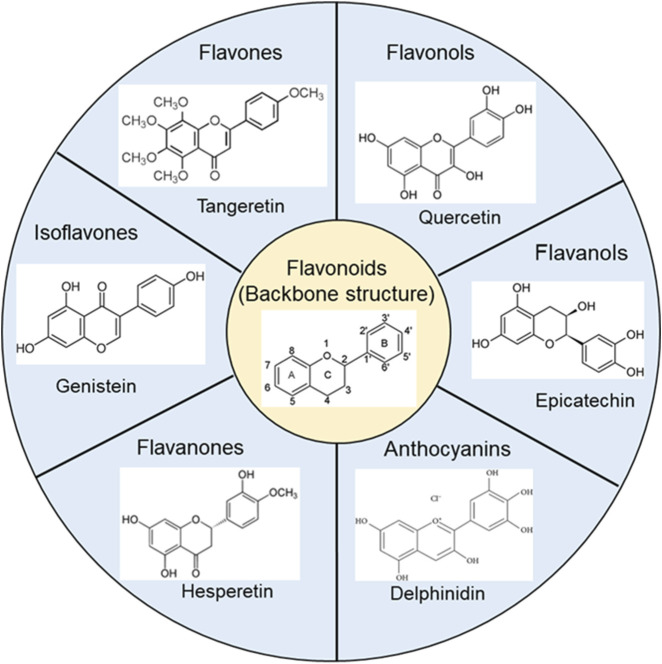
The basic backbone structure of flavonoids and representative compounds of their category.

Flavonoids are compounds extracted from many fruits, vegetables, and traditional Chinese herbal medicines. The basic backbone, classification, and chemical structures of representative flavonoids are shown in Figure 3. These compounds exhibit a variety of beneficial biological activities for human health, specially anti-inflammatory and antioxidant effects (Maleki et al., 2019; Cho et al., 2020), as well as antimicrobial and antiviral properties (Badshah et al., 2021; Cascaes et al., 2021). They have also the potential to reduce the risk of chronic diseases such as cancer (Busch et al., 2015), cardiovascular diseases (Ponzo et al., 2015; Ciumarnean et al., 2020), and neurodegenerative diseases (Devi et al., 2021). Here, we will discuss the potential anti-vasospasm effects of flavonoids through virous mechanisms.

### 3.1 Anti-vasospasm effect of flavonoids by inhibiting the SPC-induced pathway

In recent years, researchers have shown growing interest in the potential of flavonoids to inhibit abnormal contraction of VSM. To date, hesperetin and tangeretin have been found to exert significant inhibitory effects on abnormal contraction induced by SPC (Li et al., 2022; Lu et al., 2022). In addition, we discuss the anti-vasospastic effects of several other flavonoids, potentially through the attenuation of oxidative stress.

#### 3.1.1 Hesperetin

Hesperetin (the structure is shown in Figure 3), a metabolite of hesperidin, has shown promising potential in improving cardiovascular health. Animal studies have demonstrated that hesperetin can improve endothelial function (Liu et al., 2008), reduce inflammation (Wu J. et al., 2021), and exert antioxidant effects (Li et al., 2021). Furthermore, hesperetin has been found to reduce the hepatic triacylglycerol (TG) accumulation induced by 1% orotic acid (Cha et al., 2001). These findings suggest that hesperetin exerts a protective effect against cardiovascular diseases by reducing risk factors such as hypertension and dyslipidemia.

In our recent study, we observed that hesperetin effectively inhibits the abnormal contraction induced by SPC in coronary artery smooth muscle (Lu et al., 2022). Hesperetin (30 μM) markedly suppressed SPC-induced contraction, resulting in a 79.4% ± 7.4% inhibition, with an IC50 value of 13.94 μM (Lu et al., 2022). Furthermore, pretreatment with hesperetin exhibited a significant protective effect, with an inhibition rate of 80.3% ± 6.6% in response to SPC-induced contraction (Lu et al., 2022). Additionally, hesperetin was found to inhibit the translocation of Fyn and Rho-kinase from the cytoplasm to the membrane, and to attenuate SPC-induced phosphorylation of both Fyn and MLC (Lu et al., 2022). Collectively, these findings suggest that hesperetin has promising potential as a therapeutic agent for the prevention and treatment of vasospasm.

#### 3.1.2 Tangeretin

Tangeretin (the structure is shown in Figure 3), a natural compound found in citrus plants, shows promising therapeutic effects in cardiovascular diseases. Studies indicate that tangeretin exhibits anti-inflammatory (Funaro et al., 2016; Lee et al., 2016; Li et al., 2019), antioxidant (Lee et al., 2016; Li et al., 2019), and endothelial protective effects (Wu et al., 2019), which are crucial for preventing and treating cardiovascular diseases. Tangeretin has also been found to inhibit platelet activation, aggregation, and preventing thrombosis (Vaiyapuri et al., 2013). Additionally, tangeretin has been found to have lipid-lowering effects, reduce serum cholesterol levels and inhibit the expression of genes involved in lipid metabolism (Chen et al., 2021). Shiroorkar PN et al. found that tangeretin is a novel cardioprotective therapeutic agent for the treatment of sepsis-induced myocardial dysfunction (Shiroorkar et al., 2020).

A recent study by Li et al. showed that both pretreatment and posttreatment at the optimal concentration, tangeretin exhibited a remarkable inhibitory effect on the SPC-induced contraction, indicating its protective potential against cardiovascular diseases (Li et al., 2022). At the concentration of 2.5 μM, tangeretin effectively inhibited the SPC-induced contraction by 85.4% ± 8.23%, while only showing a slightly inhibitory effect (1.53% ± 2.57% inhibition) on the high K^+^-induced Ca^2+^-dependent contraction (Li et al., 2022). In addition, pretreatment with tangeretin exhibited a marked inhibitory effect on the SPC-induced abnormal contraction, with an inhibition rate of 72.51% ± 10.04%, while showing an inhibition rate of 31.89% ± 9.74% for 40 mM K^+^-induced Ca^2+^-dependent contraction (Li et al., 2022). These results suggest that tangeretin may be a potential compound for the treatment and/or prevention of vasospasm. The underlying mechanism of tangeretin’s action appears to be similar to that of hesperetin. In cultured VSM cells, tangeretin reduced MLC phosphorylation by inhibiting SPC-induced activation of Fyn and Rho-kinase, as well as their translocation from the cytoplasm to the membrane, thereby inhibiting SPC-induced abnormal contraction (Li et al., 2022).

Although inhibitory effects of hesperetin and tangeretin on SPC-induced abnormal contraction have been demonstrated in isolated tissues and smooth muscle cells, they have not yet been investigated in an animal model of vasospasm. Future in-depth studies exploring these flavonoids in this context will be crucial for advancing preclinical trials.

#### 3.1.3 Genistein

Genistein (the structure is shown in Figure 3), a natural isoflavone primarily found in soy products, has been extensively studied for its cardiovascular protective effects (Fukutake et al., 1996; Jafari et al., 2023). With its vasodilatory (Walker et al., 2001), anti-inflammatory (Goh et al., 2022), and antioxidant (Kerry and Abbey, 1998) properties, genistein has emerged as a promising candidate for the prevention and treatment of vasospasm, attracting growing interest in recent years.

As a well-known protein tyrosine kinase inhibitor, genistein significantly inhibits smooth muscle contraction, suggesting that protein tyrosine kinase plays a crucial role in regulating Ca^2+^ sensitivity of smooth muscle (Steusloff et al., 1995). Src family protein tyrosine kinases have been implicated in SPC-induced abnormal vasoconstriction (Nakao et al., 2002). It is reasonable to hypothesize that genistein may mitigate SPC-induced abnormal vascular contraction by inhibiting the activity of Src family protein tyrosine kinases. Genistein decreases RhoA activation, inhibits vascular contraction induced by U46619 and KCl, and reduces phosphorylation of MLC and MYPT1 (Thr855), suggesting that genistein may contribute to vascular relaxation and blood pressure regulation by targeting the RhoA/Rho-kinase pathway (Seok et al., 2008). Additionally, genistein’s key mechanisms of action is the enhancement of endothelial nitric oxide synthase (eNOS) activity, leading to increased NO production (Liu et al., 2004; Si et al., 2012). This, in turn, may help alleviate vasospasm and improve endothelial function.

Preclinical studies have demonstrated genistein’s vasodilatory effects in animal vascular tissues (Sato A. et al., 2000; Sun et al., 2015). Moreover, epidemiological research suggests that individuals with a diet rich in genistein-containing foods have a lower incidence of vasospastic disorders (Squadrito et al., 2003; Cruz et al., 2008). However, clinical trials specifically evaluating genistein’s efficacy in vasospasm management remain limited, underscoring the need for further investigation.

#### 3.1.4 Delphinidin

Delphinidin (the structure is shown in Figure 3) is the major anthocyanidin found in various berries and other colored fruits and possesses potent antioxidant properties (Yun et al., 2009). Studies have shown that SPC-induced NADPH oxidase (NOX) enzyme-mediated ROS generation (Shaifta et al., 2015) followed by ROS-promoted Rho-kinase activation (Jin et al., 2004; MacKay et al., 2017) may play a key role in SPC-induced vasospasm. The NOX enzyme family is a major source of ROS in various cell types (Lambeth, 2007; Zhang J. et al., 2016; Cipriano et al., 2023). A study by Lim TG et al. identified that NOX as the molecular target of delphinidin in suppressing UVB-induced MMP-1 expression in human dermal fibroblasts (Lim et al., 2013). They further found that delphinidin inhibits NOX activity, reduces ROS production, and prevents p47 (phox) translocation, thereby downregulating MKK4-JNK1/2, MKK3/6-p38, and MEK-ERK1/2 signaling pathways (Lim et al., 2013), indicating that delphinidin effectively inhibits NOX-dependent ROS generation, making it a promising candidate for therapeutic intervention in vasospasm. Delphinidin has been demonstrated to reduce ROS generation through the AMPK/NOX/MAPK signaling pathway (Chen et al., 2020). In addition, delphinidin has been demonstrated to inhibit the PDGFAB-induced release of VEGF in vascular smooth muscle cells by scavenging ROS and blocking p38MAPK and JNK pathway activation (Oak et al., 2006). These studies make delphinidin a promising candidate for therapeutic intervention in vasospasm.

### 3.2 Anti-vasospasm effect of flavonoids by inhibiting oxidative stress

Studies have shown that in order to counteract the harmful effects of oxidative stress caused by ROS, certain antioxidant enzymes play a key role in the scavenging of ROS, including superoxide dismutase (SOD), catalase (CAT), and glutathione peroxidase (GPx) (Jomova et al., 2024). SOD not only scavenges superoxide anion radicals, thereby preventing the formation of more damaging peroxynitrite, but also maintains the physiologically required level of NO (Fukai and Ushio-Fukai, 2011). Flavonoids have significant antioxidant activity, which can not only effectively scavenge ROS, but also inhibit the activity of NADPH oxidase (Luo et al., 2019), thereby reducing the production of ROS. In addition, flavonoids can also induce the activation of nuclear factor 2-related factor 2 (Nrf2) (Suraweera et al., 2020) and promote mitochondrial biogenesis (Omidian et al., 2020), thereby improving oxidative stress caused by mitochondrial dysfunction. Here, we discuss the antioxidant effects of several representative flavonoids.

#### 3.2.1 Tangeretin

Tangeretin, a polymethoxylated flavone, exhibits antioxidant biological activities. Wu et al. investigated the protective effects of tangeretin against oxygen-glucose deprivation (OGD)-induced injury in human brain microvascular endothelial cells. The results showed that tangeretin increased the SOD activity while decreasing ROS and malondialdehyde (MDA) levels (Wu et al., 2019). These effects were mediated through the suppression of the neuroinflammatory JNK signaling pathway (Wu et al., 2019). In another recent study, researchers induced brain neurotoxicity in BALB/c mice using cisplatin and investigated the potential protective effects of tangeretin. They found that ROS and MDA levels were significantly increased in cisplatin-treated brain tissue, while treatment with tangeretin reduced these levels (Cicek et al., 2024), suggesting that tangeretin has a beneficial antioxidant effect. Additionally, tangeretin activates the Nrf2 pathway (Lv et al., 2023; Peng et al., 2024), thereby enhancing the body’s endogenous antioxidant defenses and alleviating oxidative stress. Tangeretin is also reported to enhance mitochondrial biogenesis via activating the AMPK-PGC1-α pathway (Kou et al., 2018).

#### 3.2.2 Quercetin

Quercetin (the structure is shown in Figure 3), a flavonoid found in onions, apples, berries, and tea, has been extensively studied for its antioxidant and vasodilatory properties. It has been shown to effectively reduce oxidative stress by scavenging ROS and inhibiting NADPH oxidase (Zhang et al., 2020), a key enzyme responsible for ROS production in VSMCs (Drummond et al., 2011). Several studies have shown that activation of NADPH oxidase is involved in the development of coronary artery spasm and cerebral vasospasm (Kim et al., 2002; Murase et al., 2004; Saitoh et al., 2015). Quercetin mitigates vascular endothelial dysfunction in atherosclerotic mice by inhibiting the activity of myeloperoxidase and NADPH oxidase (Li J. X. et al., 2023). Furthermore, dietary quercetin has been shown to enhance NO levels and reduce ET-1 concentrations, potentially improving endothelial function (Loke et al., 2008). Quercetin can prevent ET-1-induced upregulation of NADPH oxidase and uncoupling of eNOS, thereby improving endothelial dysfunction (Romero et al., 2009). Several studies have also shown that quercetin can reduce oxidative stress response and alleviate brain damage following experimental subarachnoid hemorrhage (Dong et al., 2014; Gul et al., 2020; Jiao et al., 2023). Additionally, quercetin can also modulate mitochondrial biogenesis in various cell types (Rayamajhi et al., 2013; Li et al., 2016; Koshinaka et al., 2020). These reports suggest that quercetin has the potential to prevent cerebral vasospasm after subarachnoid hemorrhage.

#### 3.2.3 Kaempferol

Kaempferol, another flavonoid found in a variety of fruits and vegetables, has similar properties to quercetin in modulating oxidative stress and improving endothelial function (Alrumaihi et al., 2024). Recent studies highlight the link between vascular pathology, oxidative stress, and inflammation (Siti et al., 2015; Steven et al., 2019; Higashi, 2022). Kaempferol acts as a direct scavenger of ROS, reducing oxidative stress, showing great potential in the treatment of many diseases (Yao et al., 2020; Hussain et al., 2024; Yao et al., 2024). Yao et al. found that in a mouse model of vascular injury, kaempferol inhibited TNF-α and IL-6 expression and activated the Nrf2/HO-1 pathway, thereby reducing oxidative stress and inflammation and providing a protective effect on the vascular endothelium (Yao et al., 2020). A recent review summarized that kaempferol prevented neurological dysfunction in experimental models of ischemia-reperfusion and 3-nitropropionic acid-induced brain injury by inhibiting mitochondrial dysfunction, suggesting its potential use in both the prevention and post-injury treatment of brain injury (Lopez-Sanchez et al., 2024). Studies in isolated arteries have shown that kaempferol induces vasorelaxation (Xu et al., 2006; Yoon et al., 2024), which can counteract the effects of vasospasm, particularly in conditions like coronary artery spasm. Kaempferol is also reported to alleviate mitochondrial damage by reducing mitochondrial ROS production (Han et al., 2021; Lee et al., 2023).

#### 3.2.4 Apigenin

Apigenin, a flavone found in parsley, chamomile, and celery, has been reported to possess various cardioprotective effects, including modulating oxidative stress pathways involved in vasospasm (Allemailem et al., 2024). Apigenin reduces ROS levels and inhibits the activation of pro-inflammatory pathways, which are often triggered by oxidative stress in vascular tissues (Clayton et al., 2021). Apigenin induces vasodilation by promoting NO production and improving eNOS activity (Jin et al., 2009; Chen et al., 2010). Apigenin supplementation restores endothelial-dependent dilation, increases NO availability, reduces oxidative stress, and improves antioxidant enzyme expression in aged mice (Clayton et al., 2021). Zhang et al. investigated the effects of apigenin on early brain injury following SAH in rats. Apigenin administration significantly reduces brain edema, blood-brain barrier disruption, neurological deficits, and cell apoptosis by inhibiting the TLR4/NF-κB signaling pathway and upregulating tight junction proteins, suggesting its potential as a therapeutic for SAH-induced brain injury (Zhang et al., 2015). Another study by Han et al. demonstrated that apigenin treatment alleviated neurological deficits, brain edema, blood-brain barrier permeability, and cell apoptosis by reducing oxidative stress markers (ROS, MDA, myeloperoxidase (MPO)) and enhancing antioxidant activity (SOD, glutathione (GSH)), suggesting its potential as a therapeutic for SAH through its anti-oxidative effects (Han et al., 2017). By reducing oxidative stress and inflammation, apigenin can help maintain vascular homeostasis and prevent the excessive constriction of blood vessels associated with vasospasm. Furthermore, in a rat SAH model, apigenin decreased brain swelling, cell death, and neurological damage by blocking the TLR4/NF-κB pathway, which drives inflammation (Zhang et al., 2015), supporting its potential as a therapeutic agent for preventing cerebral vasospasm.

**FIGURE 4:**
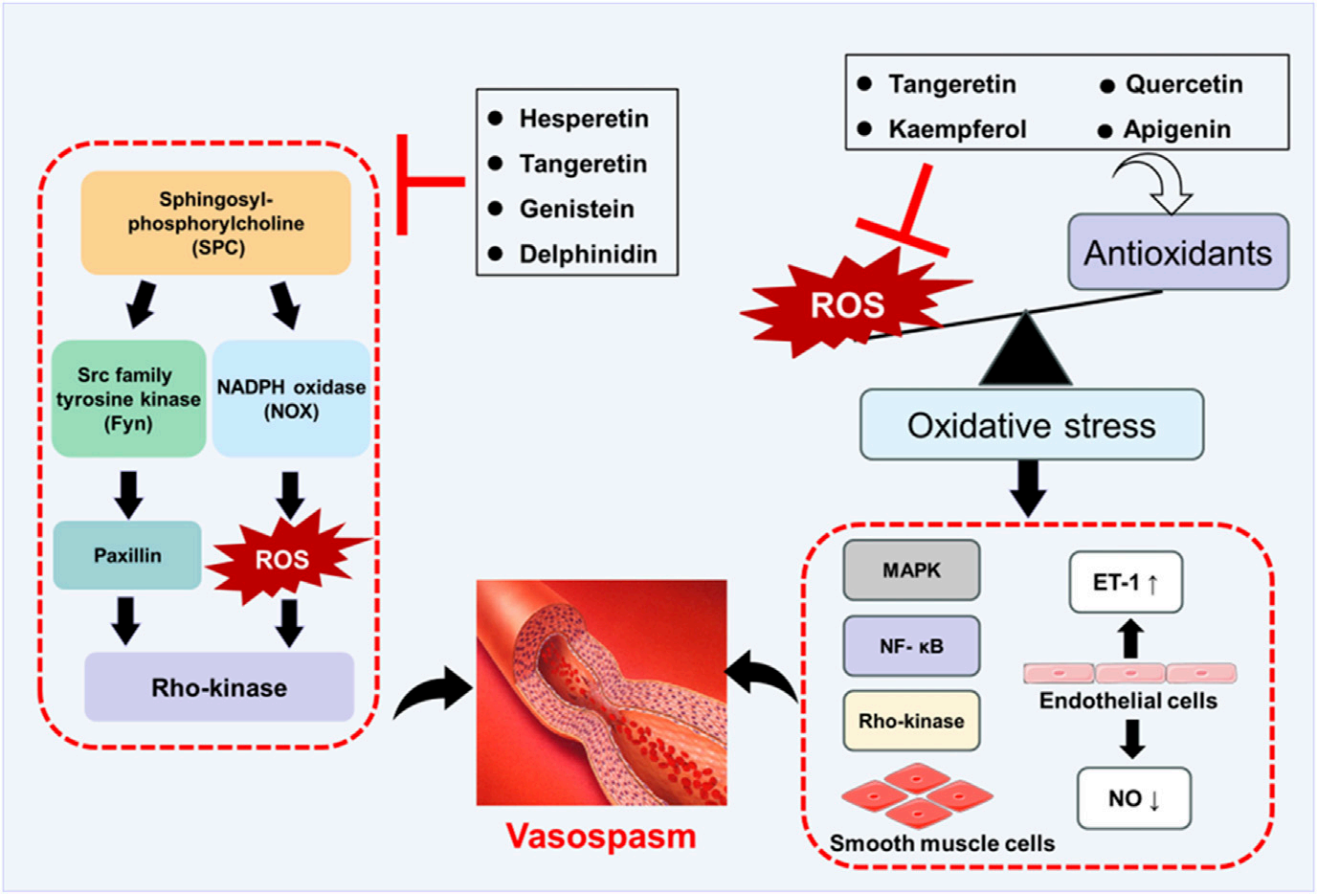
Schematic diagram of flavonoids preventing and treating vasospasm by regulating oxidative stress and SPC-induced signaling pathways.

Figure 4 shows the effects of flavonoids on oxidative stress and SPC-induced signaling pathways and their effects on vasospasm. In summary, flavonoids have shown great potential in preventing and treating vasospasm by alleviating oxidative stress, improving endothelial function, and inhibiting vascular smooth muscle contraction. Although existing studies have provided valuable experimental evidence for the use of flavonoids in vasospasm, their effects need to be further verified in preclinical studies. So far, there are few clinical trials that directly evaluate the effects of flavonoids on vasospasm. Although flavonoids are known for their powerful antioxidant and anti-inflammatory properties, their therapeutic effects are limited by low bioavailability, including low solubility, instability, rapid metabolism, and poor intestinal absorption (Thilakarathna and Rupasinghe, 2013). To overcome these challenges, several strategies have been explored, including nanocarriers, structural modifications, and advanced drug delivery systems (Nagula and Wairkar, 2019; Zhao J. et al., 2019). The pharmacokinetic properties, dose dependence, and long-term safety of flavonoids need to be further studied. Therefore, future studies should focus on exploring the effects of flavonoids in animal models and using drug delivery systems to improve their bioavailability. It is expected that clinical trials will be conducted in the near future to evaluate the effects of flavonoids on vasospasm. In addition, the synergistic effects of flavonoids combined with other drugs are also worthy of further study.

## 4 Conclusions and future perspectives

Vasospasm is an abnormal contraction of VSM, commonly occurring in coronary and cerebral arteries, which reduces blood flow and can lead to serious conditions such as myocardial and cerebral infarctions. Studies have suggested that oxidative stress and the SPC-induced signaling pathway are involved in this contraction. Flavonoids, natural compounds found in plants and traditional Chinese medicines, have shown efficacy in reducing oxidative stress and inhibiting SPC-induced pathways, indicating that they have great potential in the treatment of cardiovascular and cerebrovascular diseases associated with vasospasm. Despite the promising results of preclinical studies on these flavonoids have been demonstrated *in vitro*, additional research is required to examine their effects *in vivo*. Clinical studies are needed to validate and develop these active flavonoids for the treatment of vasospasm-associated diseases.
